# Relative adrenal insufficiency and hemodynamic status in cardiopulmonary bypass surgery patients. A prospective cohort study

**DOI:** 10.1186/1749-8090-5-26

**Published:** 2010-04-19

**Authors:** José L Iribarren, Juan J Jiménez, Domingo Hernández, Lisset Lorenzo, Maitane Brouard, Antonio Milena, María L Mora, Rafael Martínez

**Affiliations:** 1 Critical Care Department. Hospital Universitario de Canarias. Ofra s/n, La Cuesta. 38320 La Laguna. Tenerife. España; 2 Nephrology Department. Hospital Universitario Carlos Haya. Avenida de Manuel Agustín Heredia, 34, 29001 Málaga. España; 3 Biochemistry Laboratory. Hospital Universitario de Canarias. Ofra s/n, La Cuesta. 38320 La Laguna. Tenerife. España; 4 Cardiac Surgery Department. Hospital Universitario de Canarias. Ofra s/n, La Cuesta. 38320 La Laguna. Tenerife. España

## Abstract

**Background:**

The objectives of this study were to determine the risk factors for relative adrenal insufficiency in cardiopulmonary bypass patients and the impact on postoperative vasopressor requirements.

**Methods:**

Prospective cohort study on cardiopulmonary bypass patients who received etomidate or not during anesthetic induction. Relative adrenal insufficiency was defined as a rise in serum cortisol ≤ 9 μg/dl after the administration of 250 μg of consyntropin. Plasma cortisol levels were measured preoperatively, immediately before, 30, 60, and 90 minutes after the administration of cosyntropin, and at 24 hours after surgery.

**Results:**

120 elective cardiopulmonary bypass patients were included. Relative adrenal insufficiency (Δcortisol ≤9 μg/dl) incidence was 77.5%. 78 patients received etomidate and 69 (88%) of them developed relative adrenal insufficiency, (*P *< 0.001). Controlling for clinical characteristics with a propensity analysis, etomidate was the only independent risk factor associated with relative adrenal insufficiency (OR 6.55, CI 95%: 2.47-17.4; *P *< 0.001). Relative adrenal insufficiency patients showed more vasopressor requirements just after surgery (*P *= 0.04), and at 4 hours after surgery (*P *= 0.01). Pre and post-test plasma cortisol levels were inversely associated with maximum norepinephrine dose (ρ = -0.22, *P *= 0.02; ρ = -0.18, *P *= 0.05; ρ = -0.21, *P *= 0.02; and ρ = -0.22, *P *= 0.02, respectively).

**Conclusions:**

Relative adrenal insufficiency in elective cardiopulmonary bypass patients may induce postoperative vasopressor dependency. Use of etomidate in these patients is a modifiable risk factor for the development of relative adrenal insufficiency that should be avoided.

## Background

Hypothalamic-pituitary-adrenal axis activation is an essential component of the general adaptation to illness and stress and contributes to the maintenance of cellular and organ homeostasis. Relative adrenal insufficiency (RAI) is frequently diagnosed in critically ill patients [1-3], and its presence is related to poorer prognosis in patients with sepsis. This has led to recommendations for the diagnosis and management of corticosteroid insufficiency in critically ill adult patients [4]. However, the clinical impact and risk factors for RAI have not been clearly determined in cardiopulmonary bypass (CPB) patients. We hypothesized that the appearance of RAI could contribute to more complicated postoperative management in critically ill patients, increasing the use of vasoactive drugs. We aimed to assess risk factors for RAI in patients undergoing CPB, as well as their impact on postoperative vasopressor requirements.

## Methods

### Study design and patients

A prospective cohort study was performed from January to July 2007 to determine the incidence and identify risk factors associated with the development of postoperative RAI. We included 120 patients who underwent elective cardiac surgery with cardiopulmonary bypass (CPB). To avoid the confounding effect of circadian rhythm on hormone levels, all operations were performed in the morning, with general anesthesia induced between 8:30 and 9:00 am. Exclusion criteria were: history of adrenal disease, endocarditis, myocardial infarction, preoperative fever or signs of infection, surgery without CPB, emergency operations and corticoid-dependency. Postoperative care took place in a 24-bed polyvalent Critical Care Unit of University Hospital of the Canary Islands (Tenerife, Spain). Local institutional ethics committee approval was given for the study protocol, and informed consent was obtained from all patients before. This study was conducted in accordance with the provisions of the Declaration of Helsinki.

### Definition of Relative Adrenal Insufficiency (RAI) and Corticotropin test

RAI was defined as a rise in serum cortisol ≤9 μg/dl after the administration of 250 μg of corticotropin[4]. All patients underwent a 250 μg corticotropin test (Synacthene^®^; Novartis Pharma Stein AG, Stein, Switzerland) within the first four hours after surgery. Cortisol levels were measured before the test, at 30, 60 and 90 minutes after the test and finally at 24 hours after surgery. The analysis of serum cortisol was performed by radioimmunoassay (Immulite^®^; DPC Diagnostic Products, Los Angeles, CA, USA).

### Perioperative management

Anesthesia was induced and maintained by use of a standarized protocol with midazolam (0.1 mg/kg/h) combined with fentanyl (2-5 μg/kg/h) and cis-atracurium (0.06-0.18 mg/kg/h). Etomidate, a short acting intravenous anaesthetic used for the induction of general anaesthesia, was administered according to anesthetist criteria using a dosage of 0.3 mg/kg. Systemic heparinization, CPB, cardioplegic arrest and transfusion policy were performed as previously described[5]. Fluid management was carried out to achieve 8 to 12 mm Hg of central venous pressure or 12 to 15 mmHg of pulmonary artery occlusion pressure at zero positive end-expiratory pressure by infusions of crystalloids and colloids. Catecholamine support, when necessary, was used as follows: Norepinephrine was titrated to achieve a mean arterial pressure of greater or equal to 70 mmHg, and dobutamine was titrated to achieve a cardiac index of greater or equal to 2.5 L/minute per square meter. Amines were tapered off in steps of 0.02 and 1 μg/kg per minute, respectively.

### Data collection

The data collected included demographic variables, comorbidity (renal failure defined as serum creatinine >1.5 mg/dl), type of surgery and postoperative course, including relative adrenal insufficiency, norepinephrine use, ICU stay and mortality. On admission to intensive care, and after 4 and 24 hours during the postoperative period, hemodynamic data were collected using a Swan-Ganz catheter (Edwards Lifesciences LLC Irvine, CA USA). Surgical risk was calculated using the Parsonnet scale.

### Statistical analysis

Assuming an a priori 60% presentation of the event, with an accuracy of 10% in the estimate and using an asymptotic normal 95% CI, the study required the inclusion of 93 patients. In order to adjust for several confounder variables in the regression analysis, we increased the sample size to 120. Quantitative variables are reported as mean and standard deviation, or median and interquartile range as appropriate (intensive care unit length of stay). Nominal variables are reported as frequencies and percentages. Assumption of normality was tested with Kolmogorov-Smirnov test and homocedasticity with Levene test. Comparisons between groups, (patients with and without RAI) were performed using Pearson's chi-squared test or Fischer's exact test for nominal variables, and the Student's t-test or the Mann-Whitney's U test for continuous variables, as appropriate. Propensity score analysis was performed using backward binomial logistic regression analysis. The dependent variable was use of etomidate, and the independent variables were sex, age, beta-blocker treatment, diabetes, renal failure, type of intervention and Parsonnet score. Theses scores were used in a second backward logistic regression analysis. In this analysis, the dependent variable was RAI, and the independent variables were all differences in perioperative variables with a P value < 0.15, preoperative cholesterol levels, and protein levels on arrival, together with the propensity score. Bivariate associations were assessed with Spearman's rank correlation coefficient. A *P *value of less than 0.05 was considered statistically significant. SPSS 15 (SPSS Inc. Chicago, IL. USA) was used.

## Results

**Figure 1:**
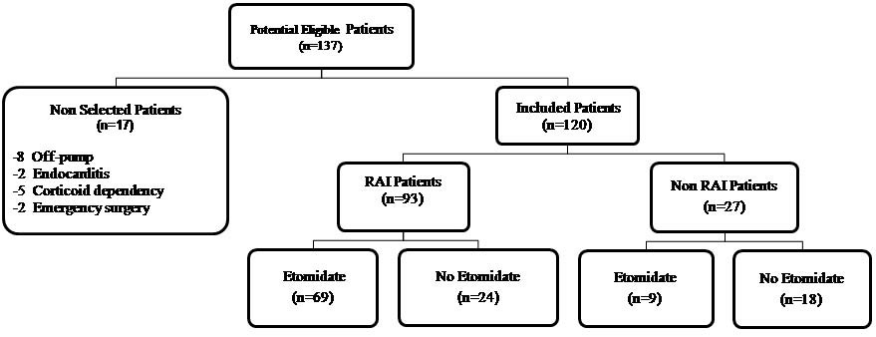
**Population study flow chart**.

One hundred and twenty from 137 consecutive eligible patients were included. Seventeen of them met criteria for exclusion (8 off-pump, 2 surgical emergencies, 2 with endocarditis, 5 corticoid-dependency), as shown in Figure 1. Demographic variables, comorbidity, medical treatments, perioperative parameters and surgical procedures of the two groups are shown in Table 1. Surgical procedures were: 65 (54.2%) coronary by-pass grafting, 39 (32.5%) valvular replacement, 12 (10%) combined surgery and 4 (3.3%) other procedures. RAI was observed in 93 (77.5%) of the whole patient sample. 78 patients received etomidate and 69 (88%) of them developed RAI, (*P *< 0.001). Logistic regression analysis including propensity score showed that the use of etomidate was significantly associated with the presence of RAI (OR 6.55, CI 95%: 2.47-17.4; *P *< 0.001) after adjusting for preoperative cholesterol levels and protein levels on admission, as shown in Table 2. The exposed attributable fraction due to etomidate was 35% (95%CI: 15-51%).

**Table 1:** Demographic variables and perioperative characteristics between groups.

	Relative adrenal insufficiency (n = 93)	No Relative adrenal insufficiency (n = 27)	*P*
Age (years)	66 ± 12	69 ± 10	*0.32*

Higher than 60 years (%)	68 (73)	25 (93)	*0.19*

Male (%)	62(67)	22(82)	*0.14*

Body mass index (kg/m^2^)	28.6 ± 4.5	27.9 ± 4.6	*0.54*

Parsonnet^a^	12.6 ± 8.6	11.7 ± 6.9	*0.64*

Hypertension (%)	56(60)	13(48)	*0.26*

Dyslipemia (%)	51(55)	11(41)	*0.20*

Diabetes (%)	30(32)	10(37)	*0.22*

Hypolipidemic drugs	63(68)	17(63)	*0.64*

ACE^b ^inhibitors (%)	26(28)	6(22)	*0.55*

Cholesterol (mg/dl)	178 ± 53	162 ± 48	*0.14*

Etomidate (%)	69(74)	9(33)	*<0.001*

Surgical procedure (%)			*0.40*
CABG^c^	49(53)	16(59)	
Valvular	29(31)	10(37)	
Combined	11(12)	1(4)	
Others	4(4)	0(0)	

Aortic clamping time (min.)	51 ± 26	55 ± 29	*0.52*

CPB^d ^time (min.)	88 ± 32	89 ± 35	*0.88*

Temperature (°C)	35.8 ± 0.7	35.7 ± 0.5	*0.81*

Protein level on ICU arrival (g/dl)	4.3 ± 0.7	4.5 ± 0.7	*0.14*

Postoperative bleeding (mL) 24 h	698 ± 229	694 ± 264	*0.96*

Re-exploration (%)	2 (2.2)	1 (3.7)	*0.96*

Length of stay in the ICU (days)^e^	3(2-6)	2(2-4)	*0.12*

Mortality (%)	3(3.2)	1(3.7)	*0.90*

Values are expressed as means and standard deviations, and frequencies and percentages. ^a^Parsonnet V et al. Circulation 1989; 79: 3-12. ^b^ACE: angiotensin converting enzyme. ^c^CABG: coronary artery bypass grafting. ^d^CPB: cardiopulmonary bypass. ^e^Values are expressed as median and 25-75^th ^percentiles.

**Table 2:** Multivariate analysis for predicting risk factors associated with relative adrenal insufficiency.

Model 1 (Unadjusted)	OR^a^	95% CI^b^	*P*
Etomidate	5.75	2.28-14.5	<0.001

**Model 2 (Adjusted)**			

Etomidate	6.55	2.47-17.4	<0.001

Cholesterol levels (mg/dl)	1.01	0.99-1.02	0.38

Protein levels on arrival (g/dl)	0.60	0.29-1.25	0.18

Propensity score	4.76	0.12-190	0.41

^a^OR: odds ratio. ^b^CI: confidence interval.

**Figure 2:**
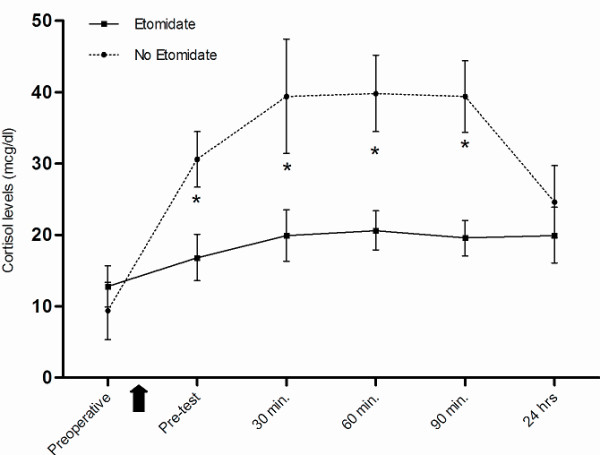
**Cortisol levels in etomidate and non-etomidate patients**. Baseline and stimulated plasma cortisol levels regarding the use of etomidate. Black arrow shows cardiac surgery. 250 μg corticotropin test was carried out at 4 hours after surgery. Values are means and 95% confidence intervals. * = *P *< 0.001 between groups.

**Figure 3:**
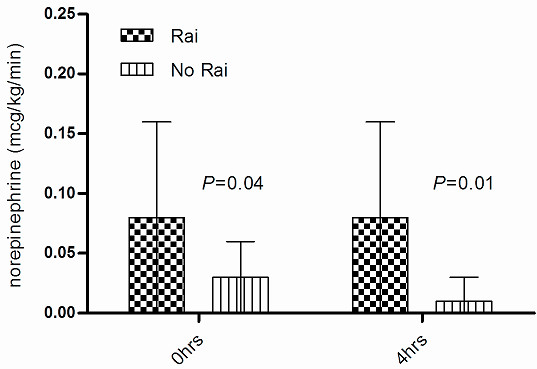
**Norepinephrine requirements**. Postoperative norepinephrine dose per group with or without relative renal insufficiency. Values are means and standard deviations.

Significantly lower cortisol levels were observed within the 4 h postoperative period (pretest) and at 30, 60, and 90 min post-test in patients who received etomidate as compared with those who did not (Figure 2). Mean arterial pressure (MAP), systemic vascular resistance index (SVRI), systolic volume index(SVI), mixed venous saturation(SvO_2_) and lactic acid were similar in both groups, although RAI patients required a higher dose of vasoactive drugs on admission to the critical care unit and during the postoperative period (4 h) (Figure 3). Likewise, a tendency to longer time requiring vasoactive drugs was also observed in RAI patients, as shown in Table 3.

**Table 3:** Hemodynamic parameters and vasoactive drug requirements between groups.

	No relative adrenal insufficiency (n = 27)	Relative adrenal insufficiency (n = 93)	*P*
MAP^a ^(mmHg) 0 hrs	82 ± 11	87 ± 16	*0.49*

MAP (mmHg) 4 hrs	77 ± 13	81 ± 10	*0.26*

MAP (mmHg) 24 hrs	83 ± 16	80 ± 10	*0.29*

SVRI^b ^(dyn-seconds·cm^-5^/m^2^) 0 hrs	2166 ± 843	2212 ± 750	*0.47*

SVRI (dyn-seconds·cm^-5^/m^2^) 4 hrs	1744 ± 392	1929 ± 402	*0.61*

SVRI (dyn-seconds·cm^-5^/m^2^) 24 hrs	1924 ± 782	1766 ± 772	*0.39*

Systolic Volume index (mL/m^2^) 4 hrs	36.1 ± 5.6	35.1 ± 4.7	0.74

Mixed venous saturation (%) 4 hrs	70 ± 10	69 ± 9	0.89

Lactic acid (mmol/L) 4 hrs	2.4 ± 0.9	2.3 ± 0.8	0.32

Norepinephrine (mcg/kg/min) 0 hrs	0.03 ± 0.03	0.08 ± 0.08	*0.02*

Norepinephrine (mcg/kg/min) 4 hrs	0.01 ± 0.02	0.08 ± 0.08	*0.01*

Norepinephrine hrs	13 ± 25	51 ± 86	*0.05*

Values are expressed as means and standard deviations. ^a^MAP: median arterial pressure. ^b^SVRI: systemic vascular resistance index.

We found an inverse relationship between pretest and post-test consyntropin cortisol values at 30, 60 and 90 min. and norepinephrine dose required in the early postoperative period (ρ = -0.20, *P *= 0.03; ρ = -0.23, *P *= 0.01; ρ = -0.25, *P *< 0.01 and ρ = -0.23, *P *= 0.01, respectively). Similarly, this also was observed in the postoperative period with maximum dose of vasoactive drugs (ρ = -0.22, *P *= 0.02; ρ = -0.18, *P *= 0.05; ρ = -0.21, *P *= 0.02; and ρ = -0.22, *P *= 0.02, respectively). Finally, no differences between the two groups were observed in postoperative bleeding, re-exploration, mortality and length of stay in the critical care unit (Table 1).

## Discussion

The major finding of our study was that the use of etomidate was an independent risk factor for RAI in patients undergoing CPB. This lead to higher requirements for vasoactive drugs in the postoperative management of these patients.

The reported prevalence of adrenal insufficiency varies widely in critically ill patients, depending on the population of patients studied and the diagnostic criteria. Recently, recommendations for the diagnosis and management of corticosteroid insufficiency in critically ill adult patients have been reported. So, RAI or critical illness-related corticosteroid insufficiency (CIRCI) is defined as inadequate cellular corticosteroid activity for the severity of the patient's illness [4]. In our study, the overall incidence of RAI was 77.5%. In agreement with previous studies describing the incidence of RAI in patients with sepsis or undergoing cardiac surgery, our patients on etomidate showed a higher rate of RAI (74%) than those who did not receive etomidate [6]. The diagnosis of RAI was based on current recommendations as previously reported, within 4 h after admission to the critical care unit[7-9].

Cardiac surgery constitutes a significantly provocative stimulus for the endogenous release of catecholamines and stress hormones. The initiation of cardiopulmonary bypass (CPB) procedure increases blood concentrations of norepinephrine, epinephrine, and cortisol[10]. In this regard, several studies have reported a rise in cortisol levels at the end of surgery that persisted in the early postoperative period, with peak values reached 4-6 hours postoperatively. This is followed by a partial return toward preoperative values at 24 hours[7-9]. In contrast, other reports have not shown variations in cortisol levels after CPB[11,12].

This response may be impaired in many critically ill patients,[1,2,13,14] including patients undergoing cardiac surgery with CPB. Henzen et al. studied adrenal function in patients who underwent CABG[15]. After administration of 1 μg of ACTH, the incidence of RAI was 25% and there were no increasing dose requirements of vasoactive drugs, but in that study no patients received etomidate, which could have influenced the results.

The only risk factor associated with RAI in our study was the use of etomidate after adjusting for confounder variables, including a control with propensity score. Adrenal suppression in humans with induction doses of etomidate has been shown in several studies,[6,16-18] suggesting suppression persisting for at least 24 h following cardiac surgery[19]. Etomidate temporarily impairs cortisol synthesis[6]. This drug has a very important role in the safe induction of unstable patients, but may impair haemodynamic status through cortisol inhibition. Notably, RAI and lower cortisol levels were related to increased need for vasoactives drugs in the early postoperative period, as well as in patients with traumatic brain injury[1]. Glucocorticoids promote the maintenance of cardiac contractility and vascular tone and decrease the production of nitric oxide, a major vasorelaxant and modulator of vascular permeability[20]. Therefore, factors affecting the release and action of cortisol may modify the hemodynamic response to stress.

Because of its cortisol-inhibiting effect, the anesthetic induction agent etomidate should be used with caution in elderly patients undergoing elective cardiac surgery[21,22]. We studied an elderly population undergoing CPB, and more pronounced RAI was observed in patients over 60 years compared with their younger counterparts. It is known that adrenal response is decreased in this population. Thus, it is plausible that the effect of etomidate could have been magnified in these patients. Future studies are needed to clarify this issue.

This study has certain limitations. Etomidate was used according to anesthetist criteria, which may have introduced a bias in the final results. We used a propensity analysis in order to elucidate whether prescription of this drug was influenced by other clinical data. Logistic regression model confirmed that etomidate use was an independent risk factor for RAI after adjusting for propensity score and other confounding variables.

In conclusion, both RAI and lower cortisol levels were associated with increased need for vasoactive drugs in elective cardiac surgery patients undergoing CPB. The use of etomidate should be minimized in elective cardiac surgery in order to decrease the hemodynamic disorders in postoperative patients.

## List of abbreviations

RAI: relative adrenal insufficiency; CPB: cardiopulmonary bypass; ICU: intensive care unit; MAP: mean arterial pressure; SVRI: systemic vascular resistance index; CIRCI: critical illness-related corticosteroid insufficiency; CABG: coronary artery bypass grafting.

## Competing interests

The authors declare that they have no competing interests.

## Authors' contributions

JLI and JJ: were responsible for the study design, data collection, processing blood samples during the study, statistical analysis, data interpretation, and drafting the manuscript.

DH: was responsible for the statistical analysis, data interpretation, and drafting the manuscript.

LL, MB, LL, SP, RP and MLM: were responsible for data collection and processing blood samples during the study and provided useful suggestions.

AM: was responsible for determination of cortisol levels.

RM: was the surgeon and was responsible for preoperative clinical and analytical data collection.

All authors read and approved the final manuscript.
